# A Au/CuNiCoS_4_/p-Si photodiode: electrical and morphological characterization

**DOI:** 10.3762/bjnano.12.74

**Published:** 2021-09-02

**Authors:** Adem Koçyiğit, Adem Sarılmaz, Teoman Öztürk, Faruk Ozel, Murat Yıldırım

**Affiliations:** 1 Department of Electrical Electronic Engineering, Engineering Faculty, Igdir University, 76000 Igdir, Turkey; 2 Department of Electronics and Automation, Vocational High School, Bilecik Şeyh Edebali University, 11230, Bilecik, Turkey; 3 Department of Metallurgical and Materials Engineering, Faculty of Engineering, Karamanoğlu Mehmetbey University, 70200, Karaman, Turkey; 4 Department of Physics, Faculty of Science, Selcuk University, 42130, Konya, Turkey; 5 Scientific and Technological Research and Application Center, Karamanoglu Mehmetbey University, 70200, Karaman, Turkey; 6 Department of Biotechnology, Faculty of Science, Selcuk University, 42130, Konya, Turkey

**Keywords:** Au/CuNiCoS_4_/p-Si device, CuNiCoS_4_, optoelectronic applications, Schottky devices

## Abstract

In this present work, CuNiCoS_4_ thiospinel nanocrystals were synthesized by hot injection and characterized by X-ray diffractometry (XRD), high-resolution transmission electron microscopy (HR-TEM), and energy-dispersive X-ray spectroscopy (EDS). The XRD, EDS, and HR-TEM analyses confirmed the successful synthesis of CuNiCoS_4_. The obtained CuNiCoS_4_ thiospinel nanocrystals were tested for photodiode and capacitance applications as interfacial layer between Au and p-type Si by measuring *I*–*V* and *C*–*V* characteristics. The fabricated Au/CuNiCoS_4_/p-Si device exhibited good rectifying properties, high photoresponse activity, low series resistance, and high shunt resistance. The *C*–*V* characteristics revealed that capacitance and conductance of the photodiode are voltage-and frequency-dependent. The fabricated device with CuNiCoS_4_ thiospinel nanocrystals can be employed in high-efficiency optoelectronic applications.

## Introduction

Recently, spinel materials have attracted great attention due their unique electronic, magnetic, optical, and gas sensing properties. Spinel compounds can be employed in data storage applications, lithium-ion batteries, gas sensors, and medical diagnostics [1–2]. Spinels have a cubic crystal structure with the general chemical formula AB_2_X_4_. In this formula, A and B are cations which can be divalent, trivalent, or tetravalent. X represents anions of a chalcogen, oxygen, or sulfur [3]. Thiospinels are one of the most interesting spinels [4–5]. These sulfur-based spinel compounds have a high potential to be used in energy applications due to their remarkable crystal, electric, thermoelectric, magnetic, and optical properties [6–7]. There are many studies on the usage of thiospinels in batteries, super-capacitors, and electrochemical reactions [8–12]. However, there are only two studies on the synthesis and application of quaternary CuNiCoS_4_ nanocrystals. The first study by Thompson is on the synthesis of CuNiCoS_4_ thiospinels [13]. The second is a study on the synthesis and photocatalytic hydrogen evolution, which was performed by our group [8]. In this study, the optical characterization results of the CuNiCoS_4_ nanocrystals have revealed that the bandgap value is suitable for optoelectronic devices. To the best of our knowledge, there is no study on the electrical properties of CuNiCoS_4_-based photodiodes.

The usage of different materials as interfacial layers in metal–semiconductor devices is a hot research topic regarding the development of more efficient metal–semiconductor devices such as photodiodes, photodetectors, and transistors [14–16]. The interfacial layer controls the current flow between metal and semiconductor and produces charge carriers under illumination [17–18]. Thiospinel CuNiCoS_4_ nanocrystals can be inserted between metal and semiconductor as interfacial layer to increase the effect of the illumination and to control electrical properties of the metal–semiconductor device.

In this work, CuNiCoS_4_ nanocrystals were successfully obtained as interlayer of Schottky diodes. The electrical characteristics of the photodiode after inserting CuNiCoS_4_ nanocrystals as interlayer between Au metal and p-Si were investigated. The aim is to obtain more powerful photodiodes by using a new type of interlayer material. XRD, HR-TEM, and SEM analyses were carried out to characterize the thiospinel CuNiCoS_4_ nanocrystals. Also, *I*–*V* and *C*–*V* measurements were performed to determine the photodiode and capacitance characteristics of the Au/CuNiCoS_4_/p-Si devices.

## Experimental

### Materials

Copper(II) acetate (CuAc_2_), nickel(II) acetate (NiAc_2_), cobalt acetate (CoAc_2_), trioctylphosphine oxide (TOPO), 1-dodecanethiol (DDT), *tert*-dodecylmercaptan (*tert*-DDT), 1-octadecene (ODE) and ethanol were purchased from Aldrich. Toluene was bought from VWR.

### Synthesis of CuNiCoS_4_ nanocrystals

CuNiCoS_4_ nanocrystals were synthesized according to the protocol described in [8]. That is, equal and stoichiometric amounts (0.125 mmol) of CuAc_2_, NiAc_2_, CoAc_2_, and 1.75 mmol TOPO were mixed with 10 mL ODE in a three-neck flask under Ar atmosphere for 30 min. Then the reaction solution was placed in a heating mantle, and the temperature was set to 210 °C. Separately, the sulfur solution was prepared by mixing 0.125 mL DDT with 0.875 mL *tert*-DDT in a glass vial, and the solution was heated up to 70 °C. When the reaction temperature reached 120 °C, the color of the reaction solution turned to black, and the sulfur solution was added into the reaction medium. The solution was heated until the synthesis temperature reached 210 °C, and it was stirred for 30 min at this temperature. At the end of synthesis time, the three-neck flask was removed from the heating mantle, and the solution was allowed to cool down to 80 °C. Finally, CuNiCoS_4_ nanocrystals were precipitated via centrifugation by adding 35 mL toluene and 5 mL ethanol.

### Fabrication of the Al/CuNiCoS_4_/p-Si device

**Figure 1:**
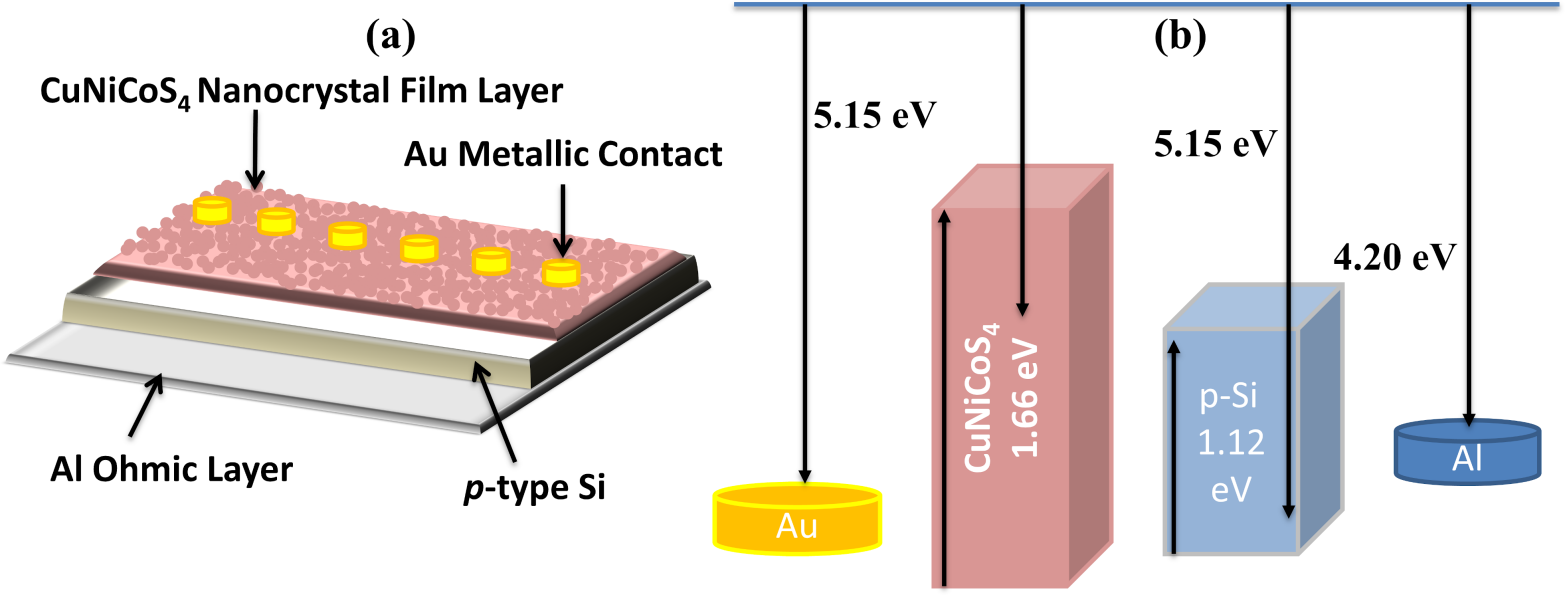
(a) Schematic illustration and (b) band diagram of the fabricated Au/CuNiCoS_4_/p-Si photodiode.

A p-type Si(100) wafer was used as substrate and as semiconductor material for the Au/CuNiCoS_4_/p-Si photodiode. First, the wafer was cut to 2 cm^2^ pieces, and acetone and propanol were used to clean the pieces in an ultrasonic cleaner. Then, the pieces were immersed in HF/H_2_O (1:10) solution for eliminating the oxide layer and impurities from the surfaces. An ohmic contact with low resistance was made by evaporation of aluminium (Al, 99.999% from Kurt J. Lesker) with a thickness of 150 nm at 5 × 10^−6^ Torr on the back side of the p-type Si substrate and subsequent thermal annealing at 400 °C for 2 min in N_2_ atmosphere. Top contacts were prepared on the ﬁlm by evaporating gold with a thickness of 150 nm at 5 × 10^−6^ Torr through a metal shadow mask (Au, 99.99% from Kurt J. Lesker). High-purity Al and Au metal contacts were thermally evaporated from a tungsten ﬁlament in a high-vacuum coating unit. Thus, the Au/CuNiCoS_4_/p-Si photodiode device was fabricated. The schematic illustration of the photodiode device and a band diagram of the junctions, with bandgaps and energy levels is shown in Figure 1. The device has a barrier at the interfacial layer between Au and p-Si. The interfacial layer might increase the barrier height between the Au and p-Si.

### Characterization

XRD patterns of the thiospinel CuNiCoS_4_ nanocrystals were recorded with a Bruker D8 diffractometer, Cu Kα radiation (λ = 0.15418 nm). A FEI TALOS F200S tunneling electron microscope (TEM) was used to understand the structural and morphological characteristics of the nanocrystals. A Zeiss-Evo SEM-EDX was employed to determine surface morphology and EDS patterns of the nanocrystals. *I*–*V* measurements were carried out on a Fytronix FY-7000 in the dark and under illumination of 20 to 100 mW in 20 mW intervals at a wavelength of 400–1100 nm. *C*–*V* measurements were carried out on a Keithley 4200 SCS.

## Results and Discussion

### Structural characterization

**Figure 2:**
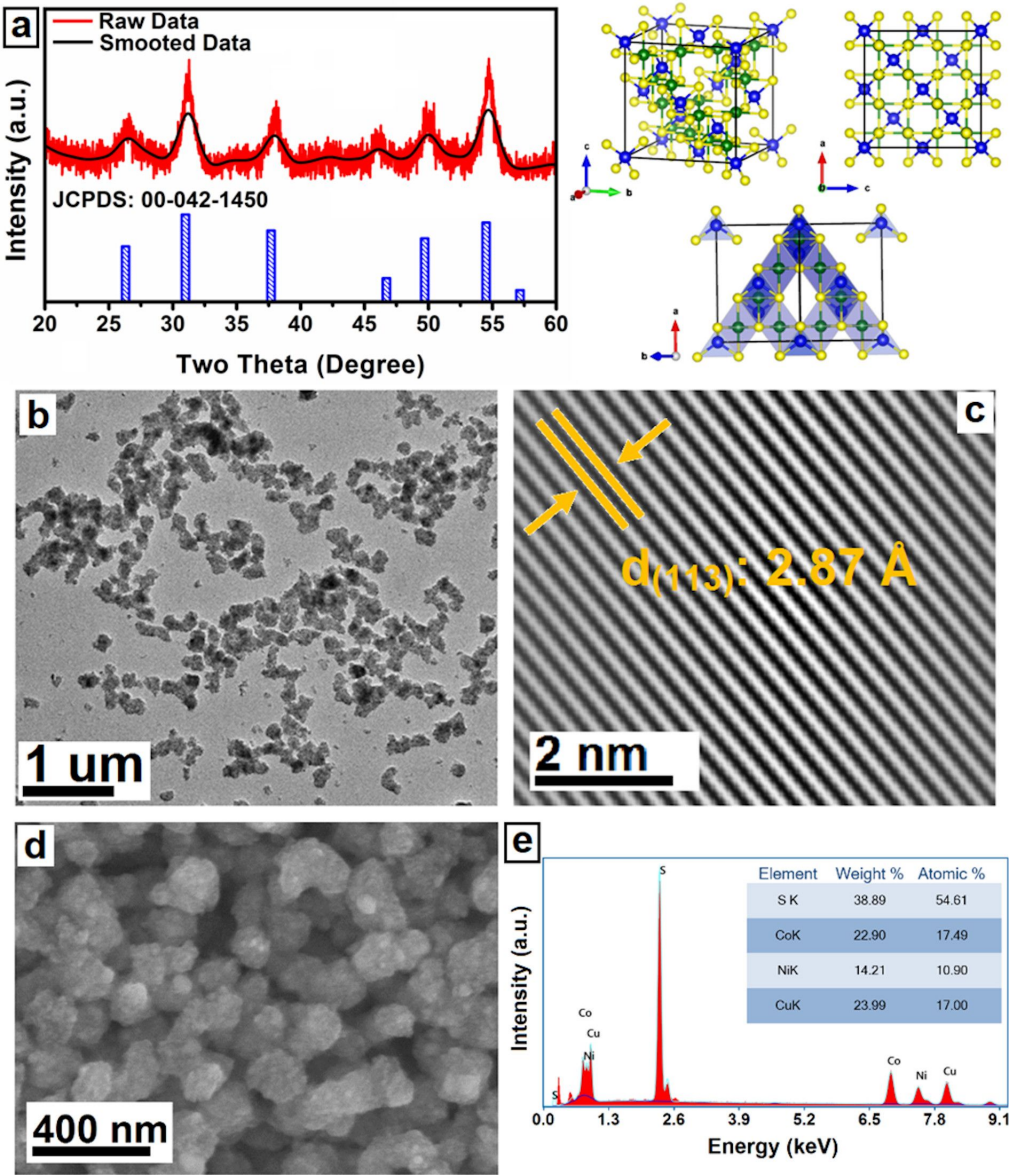
(a) XRD pattern and crystal structure model, (b) TEM image, (c) HR-TEM interplanar spacing image, (d) FE-SEM image and (e) EDS pattern of the CuNiCoS_4_ nanocrystals.

Crystal structure, phase, and purity of the produced nanocrystals were investigated by XRD analysis. The XRD pattern of the nanocrystals is shown in Figure 2a. The diffraction peaks match the cubic *Fd*−3*m* (227) space group. Furthermore, main diffraction peaks at 26.5°, 31.1°, 38.1°, 46.0°, 50.0°, and 54.7° correspond to the (022), (113), (004), (224), (115), and (044) planes, respectively (JCPDS 00-042-1450). XRD peaks of other phases were not detected, which confirms the purity of the synthesized nanocrystals.

TEM images of the CuNiCoS_4_ nanocrystals are shown in Figure 2b. Agglomerated spherical nanocrystals were formed due to magnetic effects and electrostatic and steric forces [19]. The average particle size of the CuNiCoS_4_ nanocrystals was calculated as 6.5 ± 1 nm. An interplanar spacing of *d* = 2.87 Å, corresponding to the (113) planes of the cubic crystal structure, was determined from the HR-TEM image in Figure 2c. Figure 2d shows a FE-SEM images of the agglomerated nanocrystals. The surface of the CuNiCoS_4_ nanocrystal layers is smooth and free from holes. The stoichiometric composition of the CuNiCoS_4_ nanocrystals was analyzed with EDS, and the results are given in Figure 2e. The chemical composition of the nanocrystals was determined as Cu_1.24_(NiCo)_2.08_S_4_, which is close to the theoretical composition (CuCo_2_S_4_). The morphological characterization results of the CuNiCoS_4_ nanocrystals confirmed that the nanocrystals are suitable for interfacial layers of photodiodes.

### Optical properties

**Figure 3:**
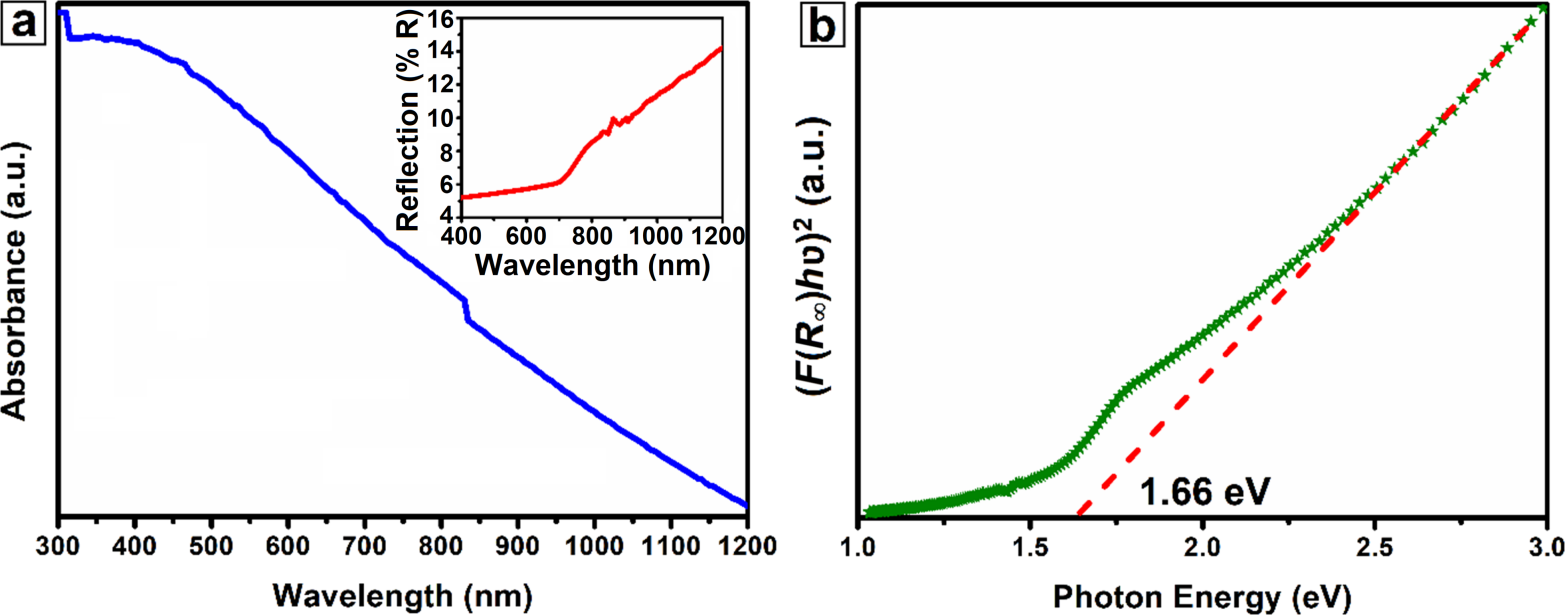
(a) Absorbance spectroscopy graph (inset figure: diffuse reflectance graph) and (b) bandgap energy diagram of CuNiCoS_4_ nanocrystals.

The optical properties of CuNiCoS_4_ nanocrystals were investigated by absorbance and diffuse reflectance spectroscopy (Figure 3). As can be seen in Figure 3a, the synthesized nanocrystals exhibit strong absorption over a broad spectrum including the ultraviolet and the near-infrared region. Furthermore, the graph of the diffuse reflectance spectroscopy is given as an inset in Figure 3a. The obtained result show that the absorption of CuNiCoS_4_ nanocrystals increases from 1200 to 300 nm, which is compatible with the absorbance result.

The optical bandgap of thiospinel CuNiCoS_4_ was calculated from the Tauc and Kubelka–Munk equations. The graph of (*F*(*R*_∞_)*h*ν)^2^ as function of the photon energy was plotted to estimate the bandgap of nanocrystals with direct band transition [8]. The bandgap was determined as 1.66 eV by extrapolating the linear portion of the band energy graph given in Figure 3b.

### Electrical properties

**Figure 4:**
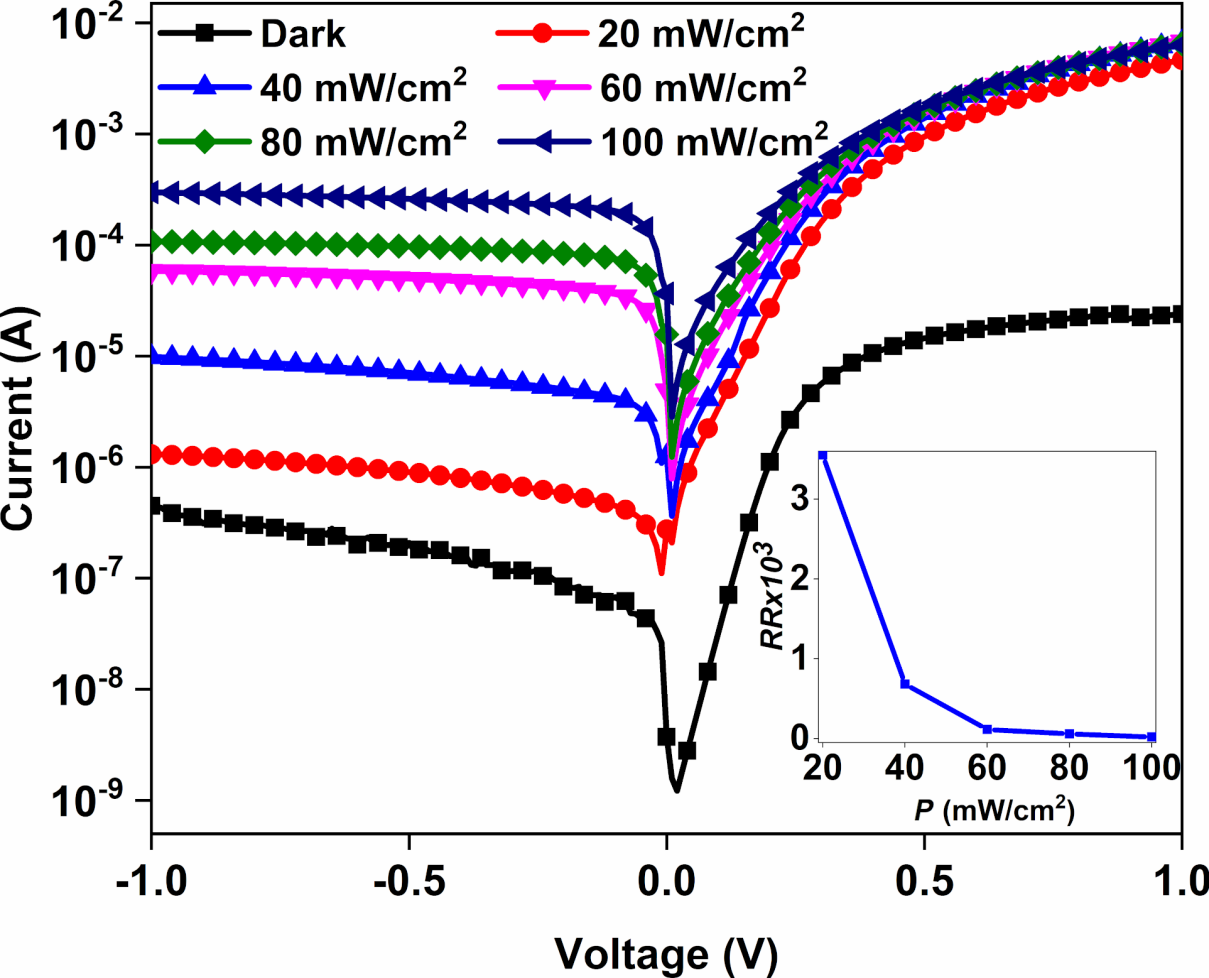
The *I*–*V* characteristics as well as *RR* changes of the Au/CuNiCoS_4_/p-Si photodiode under different illumination intensities.

In order to determine the electrical performance of the Au/CuNiCoS_4_/p-Si device, *I*–*V* measurements were performed on the photodiode in the dark and under different illumination conditions. The *I*–*V* characteristics as function of the illumination power density are shown in Figure 4. The obtained photodiode exhibited normal diode characteristics and good rectifying properties. The rectifying ratio (*RR*) values of the photodiode changed with increasing light intensity. The *RR* value was calculated as 53.25 in the dark, raising to 35.53 × 10^3^ under 20 mW·cm^−2^ illumination power density and then decreased to 21.25 with increasing illumination power density. The sharp increase of the *RR* value under 20 mW·cm^−2^ illumination power density and the slow decrease with increasing light intensity can be attributed to the increasing of the current at the interface at forward biases [20]. The *RR* value as function of the illumination power density is displayed in the inset of Figure 4.

The fabricated Au/CuNiCoS_4_/p-Si photodiode exhibited almost linear growth of the current values with increasing illumination power density due to enhancing number of charge carriers at the interface at reverse biases. A photocurrent value 1000 times higher than the dark current was obtained, owing to the existence of the interfacial CuNiCoS_4_ layer and the semiconductor p-Si [21–22]. The increase of the current at zero bias voltage from dark to 20 mW/cm^2^ light power illumination intensity can be attributed to the increasing number of carriers at the interface of the Au/CuNiCoS_4_/p-Si device due to illumination. The depletion region between the metal and semiconductor can cause a current in the device. Furthermore, the CuNiCoS_4_ layer can also increase the current at zero bias voltage due its high absorption of the solar light. The CuNiCoS_4_ layer has a suitable bandgap value for the solar spectrum and can be used for optoelectronic applications, such as photodiode or photodetector, due to its good response to increasing illumination power densities [8].

The diode parameters, such as ideality factor (*n*), series resistance (*R*_s_), and barrier height (ϕ_b_), of the fabricated Au/CuNiCoS_4_/p-Si device provide information to understand electrical characteristics. These parameters can be extracted from the *I*–*V* measurements by various techniques such as thermionic emission theory and the Norde method. The current (*I*) is calculated using the following equation from thermionic emission theory:

[1]
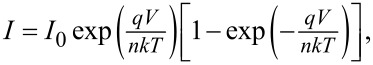


where *I*_0_ is the saturation current, which is calculated as follows:

[2]
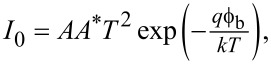


where *k* and *q* are the Boltzmann constant and the electron charge, respectively. *T*, *A*, and A* are temperature, diode area (here *A* = 7.85 × 10^−3^ cm^2^), and Richardson constant (32 A·cm^−2^·K^−2^ for p-type Si), respectively. Equation 2 can be used to calculate ϕ_b_ from the saturation current value of the *I*–*V* curve of the Au/CuNiCoS_4_/p-Si photodiode. *I*_0_ was determined as 6.58 × 10^−10^ A for the Au/CuNiCoS_4_/p-Si device from the (ln *I*)–*V* plot for the second linear region between 0.25 and 0.98 V, and the ϕ_b_ value was calculated from that. The slope of the second linear region of the *I–V* curve helps to calculate the value of *n* for *V* ≥ 3*kT*/*q* by the following equation:

[3] $$n=\frac{q}{kT}\left(\frac{\text{d}V}{\text{d}\ln I}\right),$$

and ϕ_b_ is obtained from the equation:

[4] $$\phi_{\text{b}}=\frac{kT}{q}\ln\left(\frac{A^{*}AT^{2}}{I_{0}}\right).$$

**Figure 5:**
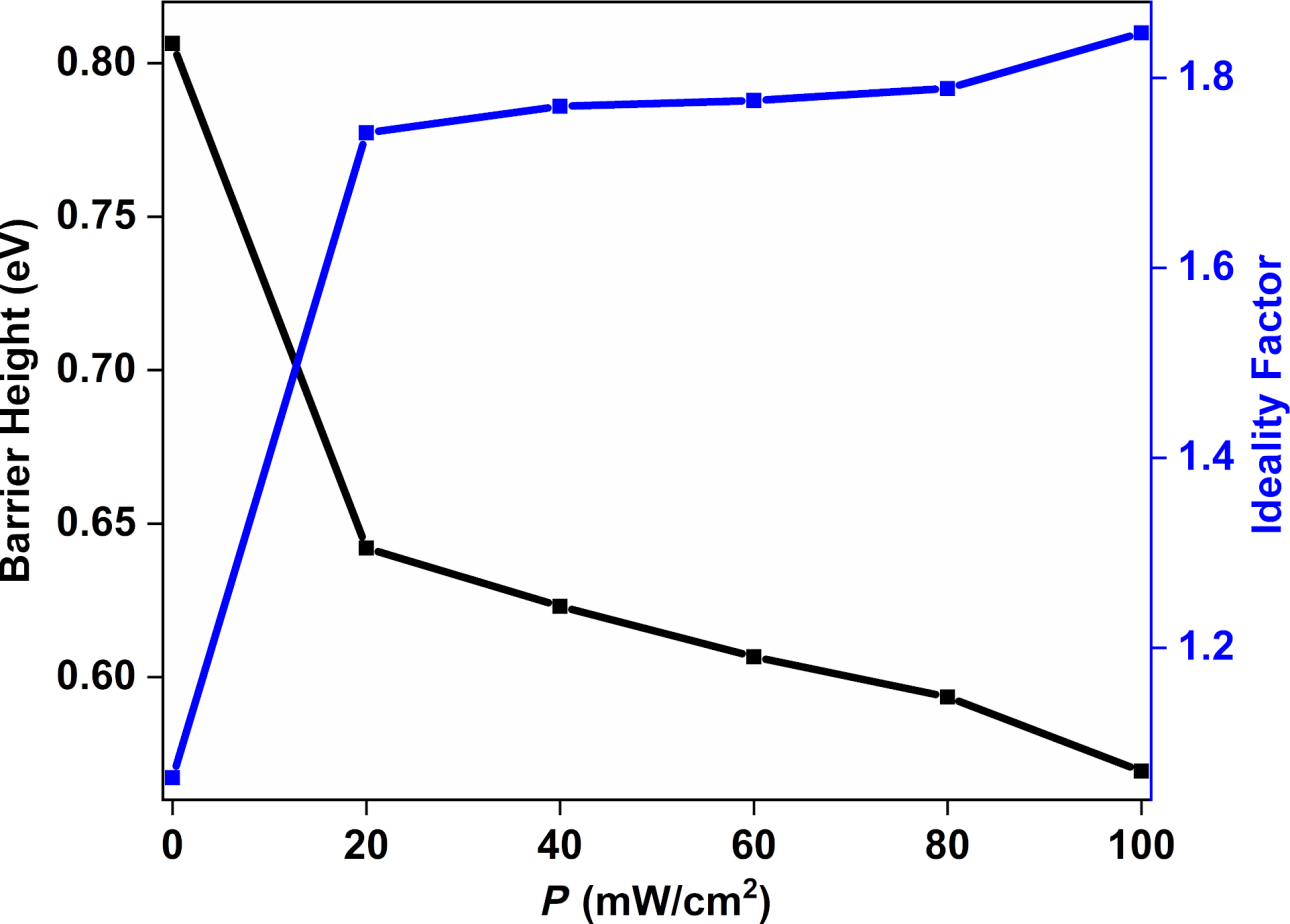
The values of *n* and ϕ_b_ of the Au/CuNiCoS_4_/p-Si photodiode as functions of the illumination power density.

The calculated *n* and ϕ_b_ values as functions of the illumination power density is displayed in Figure 5 and also listed below in Table 1. While the ideality factor value increased from 1.06 to 1.85, the barrier height value decreased from 0.81 to 0.57 eV with increasing illumination power density. An *n* value close to unity was obtained in the dark. The increasing light intensity caused a change of the charge carrier density distribution, and thus the ideality factor value increased [23–24]. The ideality factor close to unity can be attributed to a homogenous interfacial layer of CuNiCoS_4_.

The interfacial layer can passivate the dangling bonds and decrease the density of interface states. Thus, the ideality factor values approaches unity [25–26]. The decrease of ϕ_b_ values with increasing light power density can be attributed to charge carriers with high energy passing easily the barrier between metal and semiconductor. Also, more charge carriers are generated with increasing light power, and thus diffusion of the charges from the barrier occurs [27].

The junction resistance (*R*_j_) is another diode parameter to evaluate the fabricated Au/CuNiCoS_4_/p-Si photodiode, and it can be determined from *I*–*V* characteristics [28]. *R*_j_ contains two components: shunt resistance (*R*_sh_) due to contact of the metal–semiconductor interface and series resistance (*R*_s_) owing to interfacial layers [29]. *R*_j_ can be calculated as follows:

[5] $$R_{\text{j}}=\frac{\partial V}{\partial I}.$$

**Figure 6:**
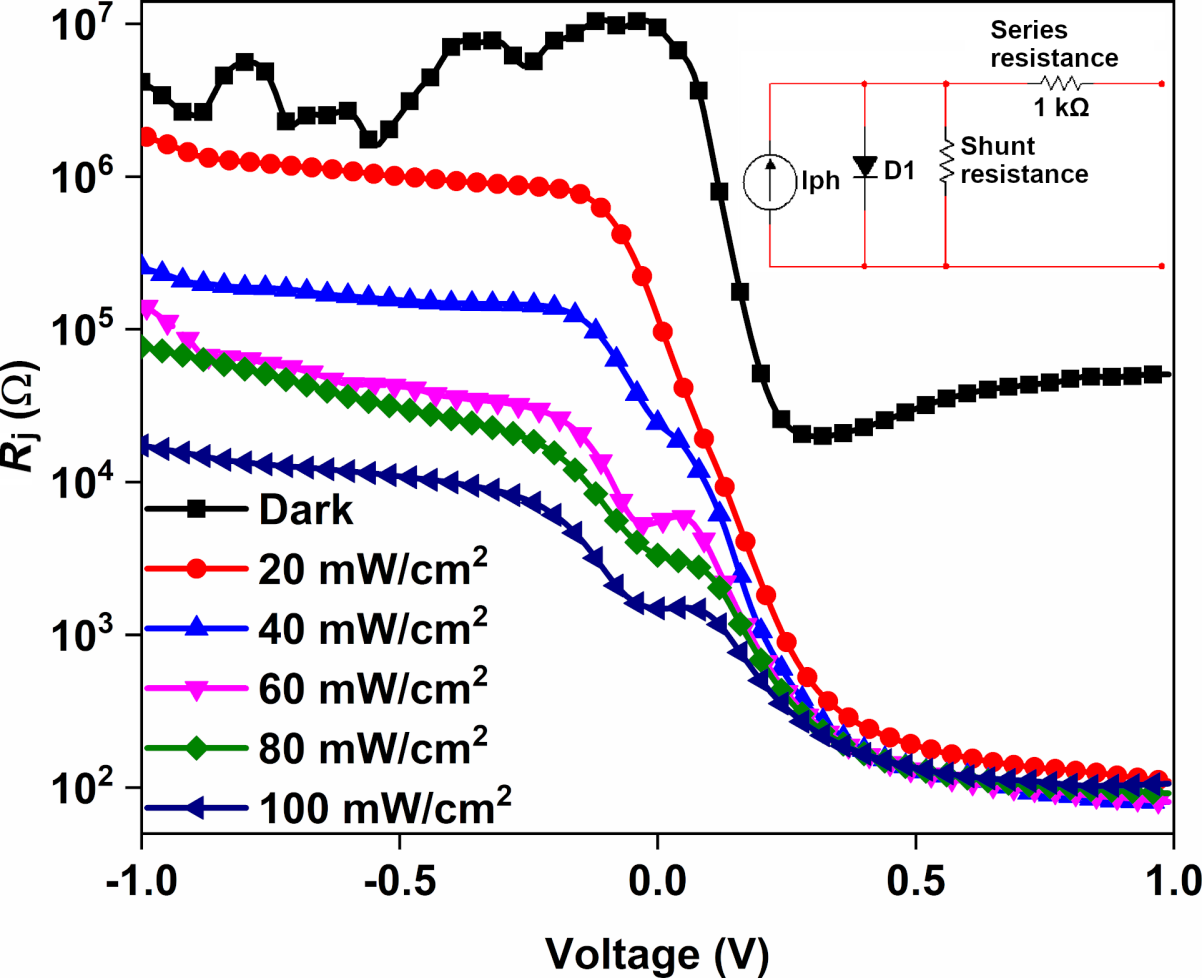
*R*_j_–*V* plots (with equivalent circuit of serial and shunt resistance in the inset) of the Au/CuNiCoS_4_/p-Si photodiode for different illumination power densities.

The *R*_j_–*V* plots of the Au/CuNiCoS_4_/p-Si photodiode are given in Figure 6 for different illumination power densities. While the *R*_sh_ values are determined from reverse-bias *R*_j_ values, the *R*_s_ values are obtained at forward biases. The *R*_sh_ value of the photodiode was of the order of 10^7^ Ω. The *R_s_* value was determined as of the order of 10^4^ Ω in the dark. The obtained *R*_s_ and *R*_sh_ values are listed below in Table 1. Both *R*_s_ and *R*_sh_ decreased with increasing illumination power density. The obtained results highlight that the photodiode can be used for optoelectronic applications [30]. The slightly increasing *R*_s_ value in the dark can be attributed to a constant current with increasing voltage due to the interface states of the device.

Another method to determine the barrier height and series resistance values is the Norde technique. The Norde function is defined as follows [31]:

[6] $$F\left(V\right)=\frac{V}{\gamma }-\frac{kT}{q}\ln\left(\frac{I\left(V\right)}{AA^{*}T^{2}}\right),$$

where γ is the closest integer higher than *n*. *I*(*V*) is the voltage-dependent current. ϕ_b_ and *R*_s_ are obtained according to the following equations:

[7] $$\phi_{\text{b}}=F\left(V_{0}\right)+\left[\frac{V_{0}}{\gamma }-\frac{kT}{q}\right]$$

**Figure 7:**
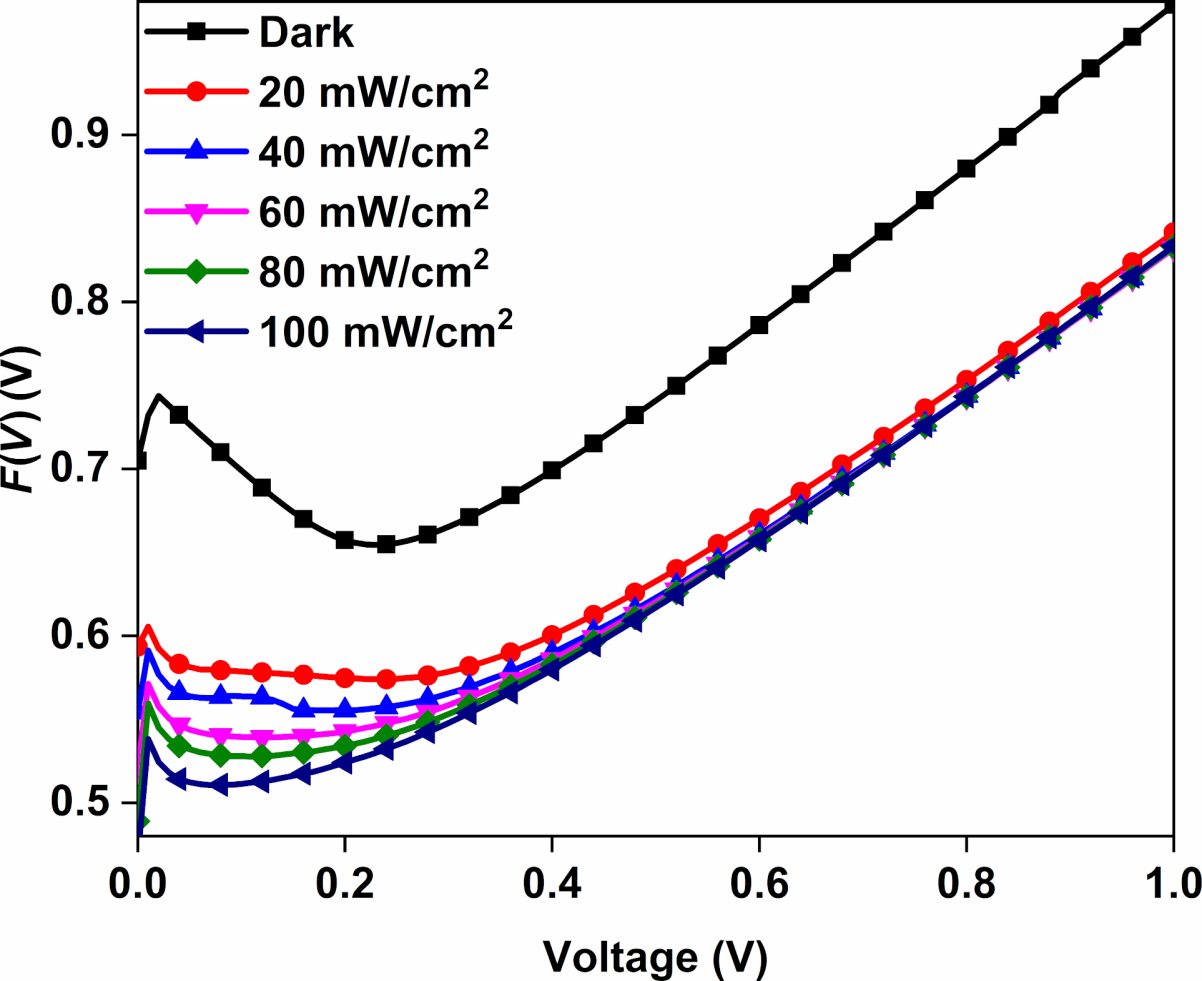
*F*(*V*)–*V* plots of the Au/CuNiCoS_4_/p-Si photodiode for various illumination power densities.

[8] $$R_{\text{s}}=\frac{\gamma -n}{I}\frac{kT}{q},$$

where *V*_0_ is the minimum voltage corresponding to *F*(*V*). *F*(*V*) versus *V* plots of the Au/CuNiCoS_4_/p-Si photodiode are shown in Figure 7 for different illumination power densities. The calculated ϕ_b_ and *R*_s_ values are given in Table 1. The *F*(*V*)–*V* plots represent normal Norde function plots, and the *F*(*V*) values decreased with increasing light power, especially in the low-voltage region due to the increasing photocurrent. The obtained values of ϕ_b_ and *R*_s_ are in good agreement with the ϕ_b_ and *R*_s_ values derived from thermionic emission. Small differences of the ϕ_b_ and *R*_s_ values can be attributed to the used approximation [29].

The responsivity and specific detectivity are other important parameters of a photodiode or photodetector. While the responsivity represents the response to the incident light, the specific detectivity represents the inverse of the noise equivalent power [32]. Both responsivity and specific detectivity increased with increasing illumination power density and confirmed the good performance of the fabricated Au/CuNiCoS_4_/p-Si photodiode. This behavior was studied and discussed in the literature for various organic interlayers [33–34]*.*

**Table 1:** Diode parameters of the Au/CuNiCoS_4_/p-Si photodiode.

Condition	*I*_0_ (A)	*n* (*I*–*V*)	ϕ_b_ (*I*–*V*) (eV)	ϕ_b_ Norde (eV)	*R*_sh_ (kΩ)	*R*_s_ (kΩ)	*R*_s_ (Norde) (kΩ)	Responsivity (A/W)	Detectivity (Jones)

Dark	6.58 × 10^−10^	1.06	0.81	0.83	7934	19.57	9.12	—	—

20 mW	3.79 × 10^−7^	1.74	0.64	0.66	876	0.14	0.13	4.87	9.99 × 10^11^

40 mW	7.89 × 10^−7^	1.77	0.62	0.62	157	0.13	0.15	6.99	1.39 × 10^12^

60 mW	1.49 × 10^−6^	1.78	0.61	0.58	33.7	0.14	0.21	13.97	2.68 × 10^12^

80 mW	2.47 × 10^−6^	1.79	0.59	0.56	22.1	0.14	0.19	12.15	2.33 × 10^12^

100 mW	6.29 × 10^−6^	1.85	0.57	0.52	7.67	0.13	0.15	10.48	2.11 × 10^13^

**Figure 8:**
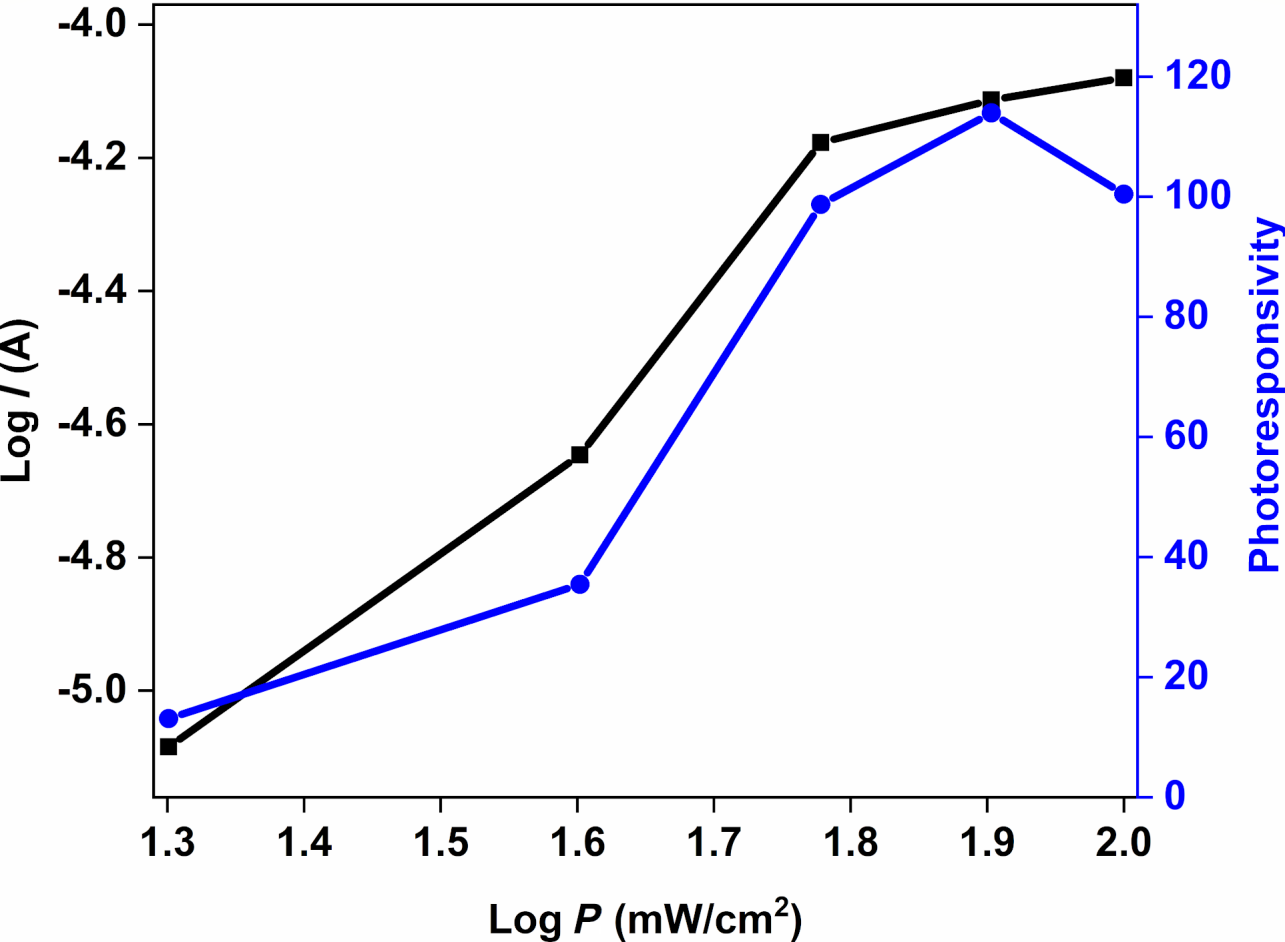
Graphs of log *I* and photoresponsivity as functions of log *P* of the Au/CuNiCoS_4_/p-Si photodiode for various illumination power densities.

The graphs of log *I* and photoresponsivity as functions of log *P* of the Au/CuNiCoS_4_/p-Si are given in Figure 8. The photoconducting behavior of the diodes is described by the relation *I* = *BP^m^*, where *I* is the photocurrent, *B* is a constant, *P* is the illumination intensity, and *m* is the illumination coefﬁcient. The value of *m* of the diode is obtained from the slope of the log *I*-vs-log *P* plot. The *m* value determines the type of photoconducting mechanism of the diode. If *m* is between 0.5 and 1, the photoconducting mechanism is related to trap levels. Values higher than unity suggest that the photoconducting mechanism is due to unoccupied trap levels. The *m* value of the diode was found to be 1.5 from plots of log *I* vs log *P* in Figure 8. The diode exhibited a linear photoconducting mechanism. Both log *I* and photoresponsivity increased almost linearly with increasing illumination power density. The linear increase of the photocurrent can be attributed to the photoconductive mechanism of the Au/CuNiCoS_4_/p-Si. According to the changes of photoresponsivity and current with increasing illumination power density, the Au/CuNiCoS_4_/p-Si heterostructure is a promising candidate for optoelectronic applications [35].

**Figure 9:**
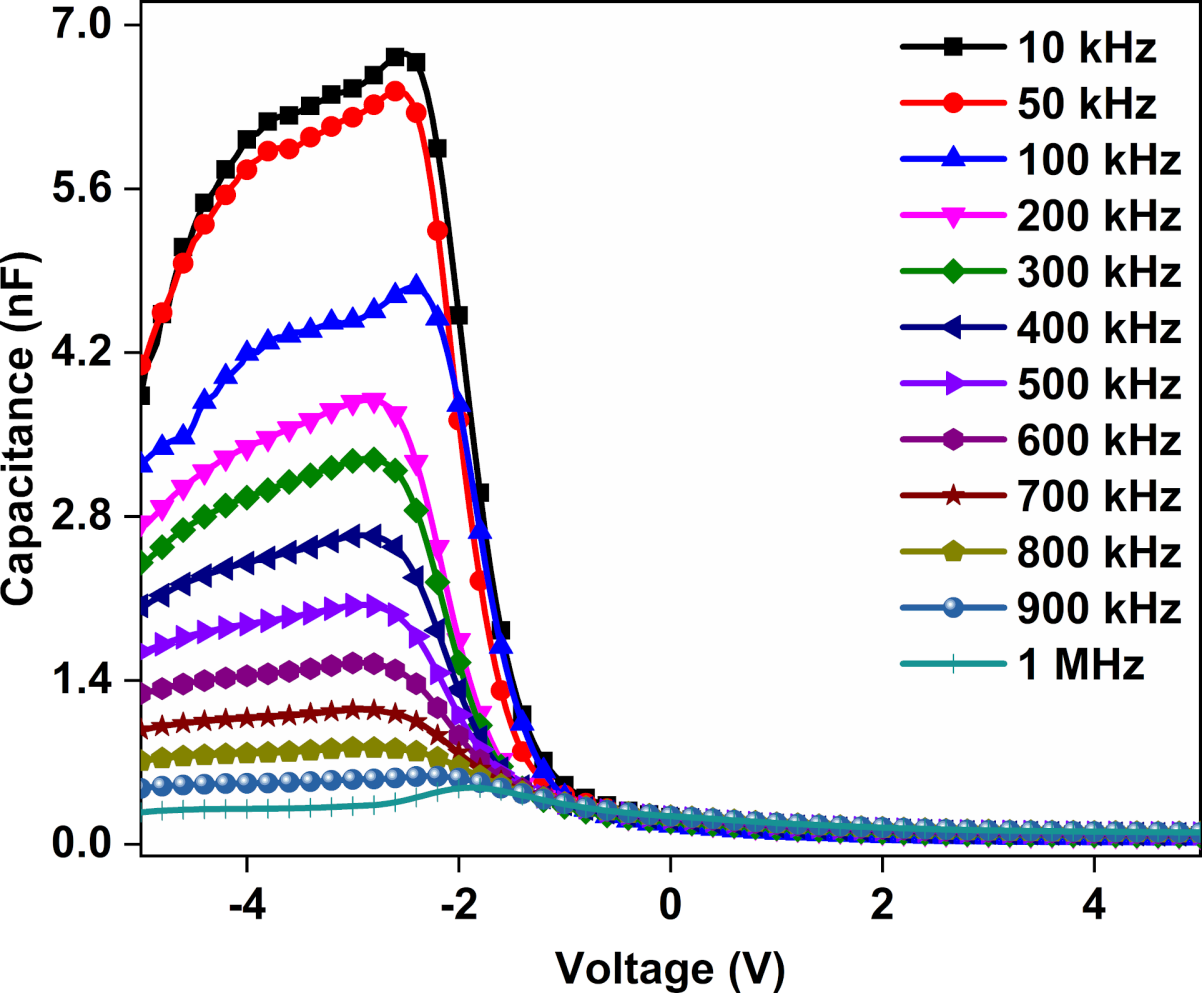
*C*–*V* graphs of the Au/CuNiCoS_4_/p-Si photodiode for increasing frequencies.

The fabricated Au/CuNiCoS_4_/p-Si photodiode was studied with *C*–*V* and *G*–*V* measurements to understand the frequency response and obtain some other electrical parameters. The *C*–*V* graphs of the Au/CuNiCoS_4_/p-Si photodiode are given in Figure 9 for the frequency range of 10 kHz to 1 MHz at voltages from −5 to 5 V. The *C*–*V* characteristics revealed that the capacitance values did not change in accumulation and depletion regions when changing voltage and frequency but increased suddenly in the inversion region and exhibited peaks. The intensity of the peaks decreased at higher frequencies, and the peak positions shifted when changing the voltage. The presence of the peaks highlights the existence of a particular distribution of the interface states or the CuNiCoS_4_ layer [36–37]. The decrease of the capacitance values with increasing frequency can be attributed to interface states [38–39].

**Figure 10:**
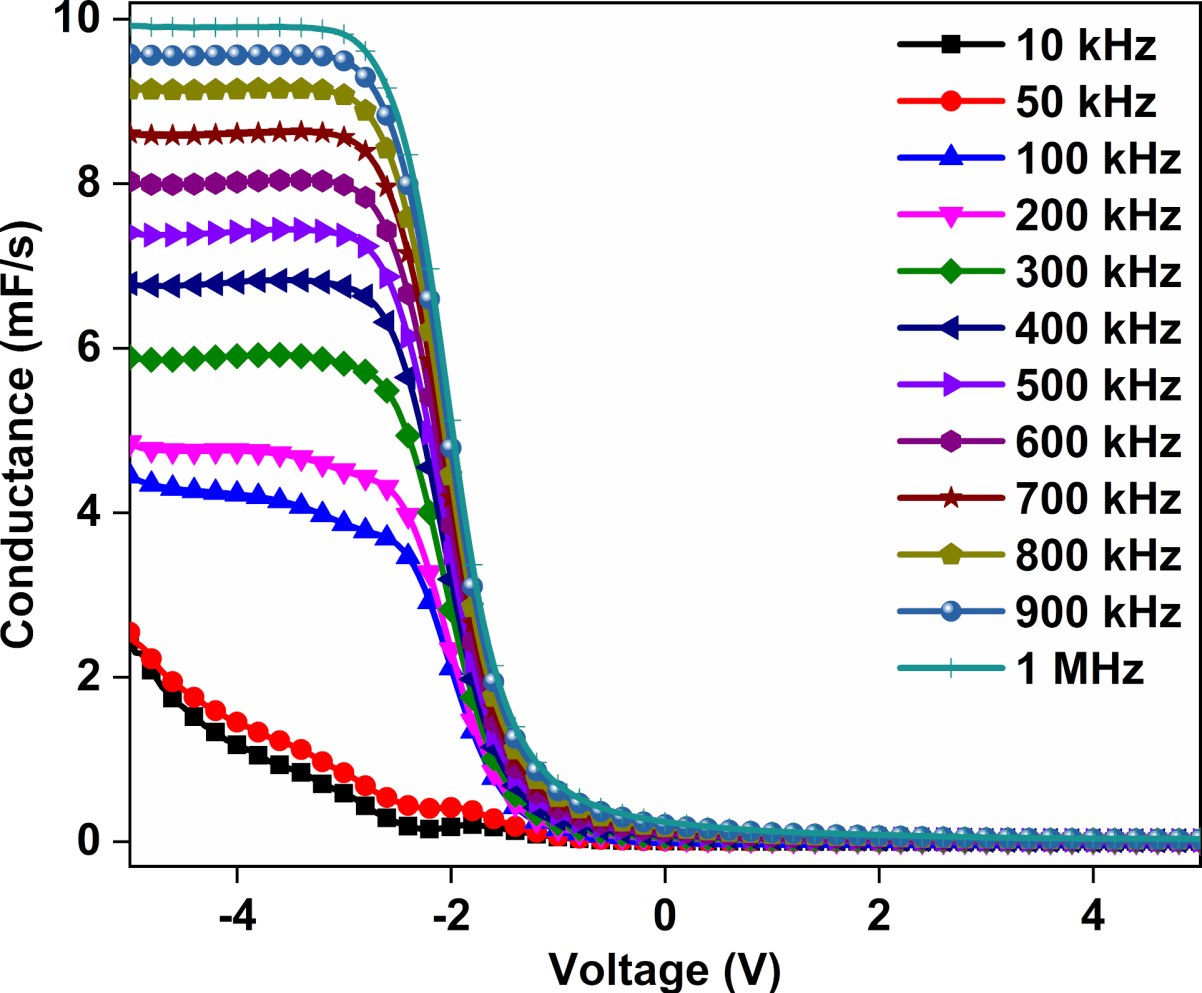
*G*–*V* graphs of the Au/CuNiCoS_4_/p-Si photodiode for increasing frequencies.

The conductance–voltage (*G*–*V*) graphs of the Au/CuNiCoS_4_/p-Si photodiode for different frequencies are displayed in Figure 10. Again, the conductance values did not change in the accumulation and depletion regions. However, the conductance values change in the inversion region. The conductance values for frequency values higher than 100 kHz increased suddenly and stayed almost constant towards regions of higher inversion. Furthermore, the conductance values increased with increasing frequency in the inversion region due to effect of the interface states in the photodiode [40–41]. The sudden increase of the conductance values with increasing reverse bias can be attributed to the applied electric field causing a change of the behavior of the semiconductor.

**Figure 11:**
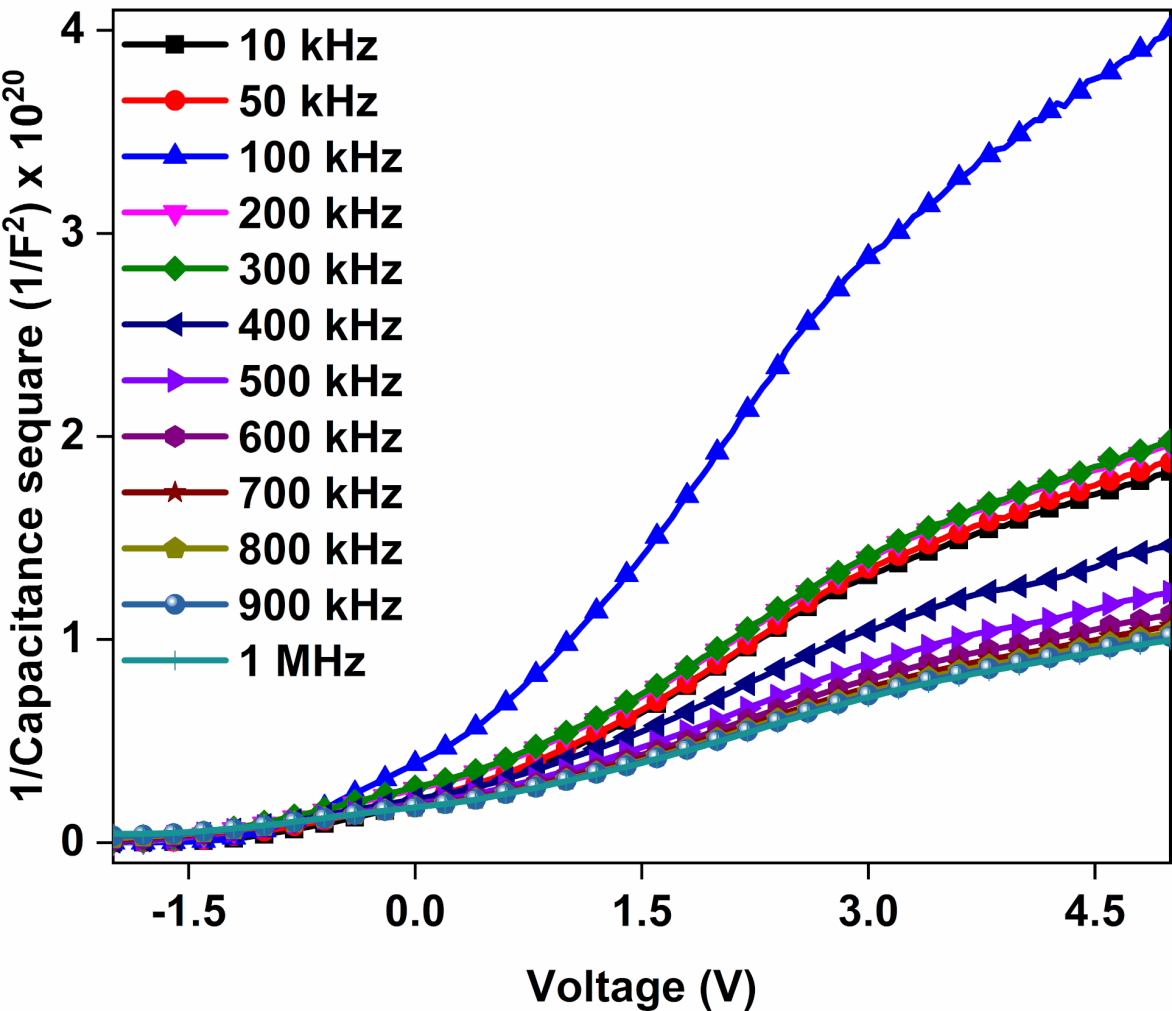
*C^−^*^2^–*V* plots of the Au/CuNiCoS_4_/p-Si photodiode for increasing frequencies.

*C^−^*^2^–*V* graphs of the Au/CuNiCoS_4_/p-Si device are displayed in Figure 11 for various frequencies. The graphs exhibit sometimes straight lines and sometimes deviations from linearity due to a non-homogenous interfacial layer of CuNiCoS_4_ [42]. Various electrical parameters, such as Fermi energy level (*E*_F_), barrier height (ϕ_b_), maximum electric field (*E*_m_), depletion width (*W*_d_), and doping concentration of acceptor atoms (*N*_a_) and interface states (*N*_ss_), were calculated from the *C^−^*^2^–*V* graphs and listed in Table 2 for different frequencies [43].

**Table 2:** Electrical parameters calculated from *C^−^*^2^–*V* plots of the Au/CuNiCoS_4_/p-Si photodiode for different frequencies.

*f* (kHz)	*N*_a_ (10^15^ cm^−3^)	*R*_s_ (Ω)	ϕ_b_ (eV)	*E*_F_ (eV)	*E*_m_ (×10^4^ V/cm)	*W*_d_ (×10^−5^ cm)	*N*_ss_ (×10^10^ eV^−1^· cm^−2^)

10	7.822	364.58	1.009	0.138	4.545	3.881	16.693
50	7.605	357.63	1.029	0.138	4.530	3.977	5.140
100	4.670	348.92	1.157	0.151	3.769	5.380	9.702
200	6.972	326.03	1.067	0.141	4.424	4.235	5.576
300	6.823	307.33	1.084	0.141	4.415	4.317	5.357
400	9.034	288.13	1.093	0.134	5.128	3.787	5.295
500	10.181	283.12	1.100	0.131	5.475	3.587	5.353
600	11.058	276.77	1.107	0.129	5.735	3.459	5.446
700	11.522	270.16	1.126	0.128	5.914	3.422	5.570
800	11.805	265.39	1.127	0.127	5.992	3.384	5.719
900	11.877	262.12	1.139	0.127	6.046	3.393	5.912
1000	12.102	264.89	1.154	0.126	6.151	3.388	6.168

While the *N*_a_, *E*_m_ and ϕ_b_ values usually increase, the values of *E*_F_, *R*_s_, and *N*_ss_ decrease with increasing frequencies. The *W*_d_ values increased up to 100 kHz with increasing frequency and then decreased towards 1000 kHz. The decreasing of *R*_s_ with increasing frequency can be attributed to an increase of the conductivity of the photodiode. The *N*_ss_ values for the Au/CuNiCoS_4_/p-Si photodiode are acceptable for optoelectronic devices [44].

**Figure 12:**
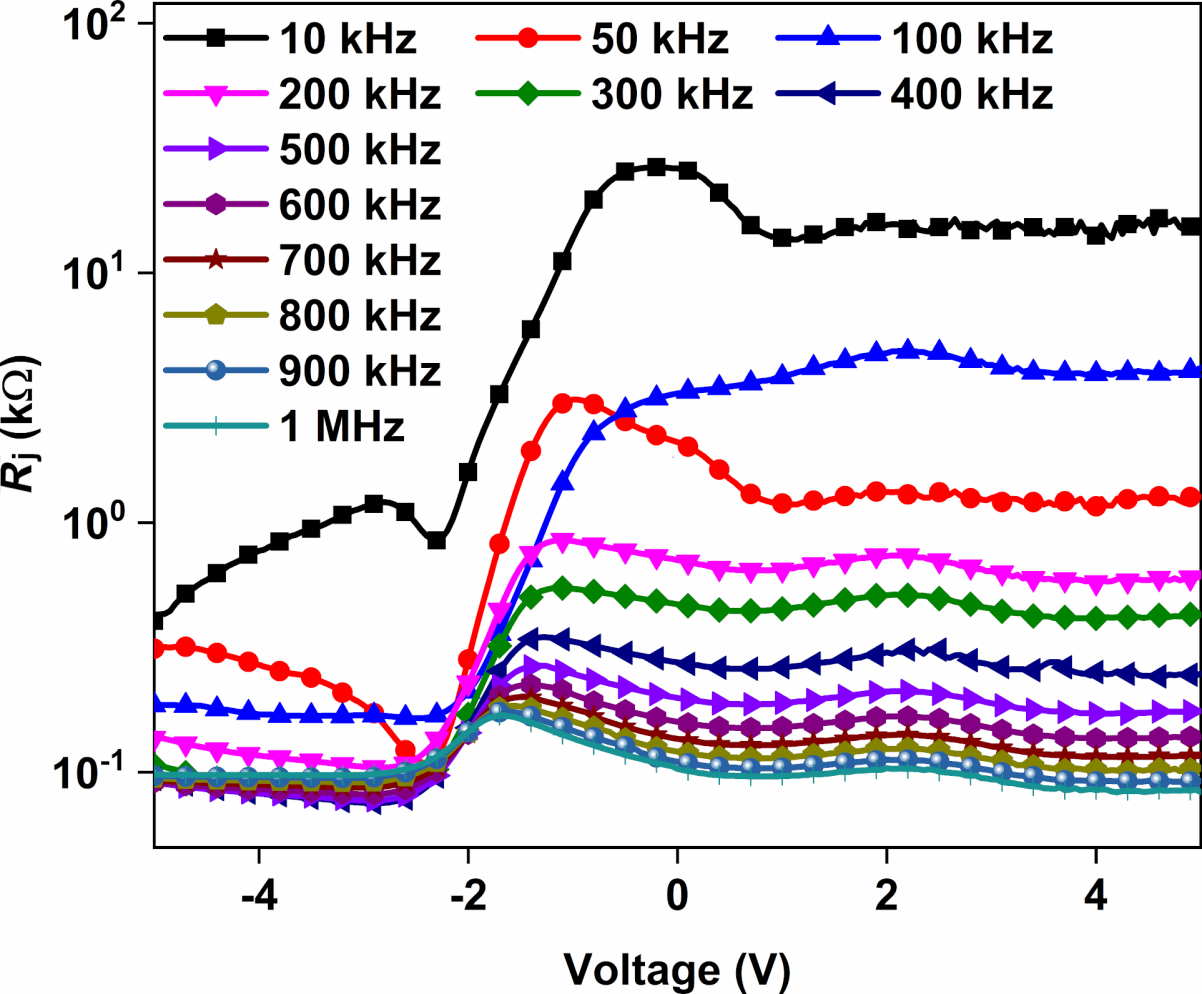
The *R*_i_–*V* plots of the Au/CuNiCoS_4_/p-Si photodiode for changing frequency.

The plots of resistance as function of the voltage (*R*_i_–*V*) of the Au/CuNiCoS_4_/p-Si photodiode are given in Figure 12 for different frequencies and voltages. The *R*_i_ values usually decrease with increasing frequency due to the increasing conductance of the Au/CuNiCoS_4_/p-Si photodiode. The frequency-dependent *R*_i_ profiles exhibit peaks in the depletion region because of interface states [45]. The peak positions shifted towards the inversion region. Furthermore, the *R*_i_ values almost did not change in the inversion region but suddenly increased in the depletion region and stayed almost constant in the accumulation region.

**Figure 13:**
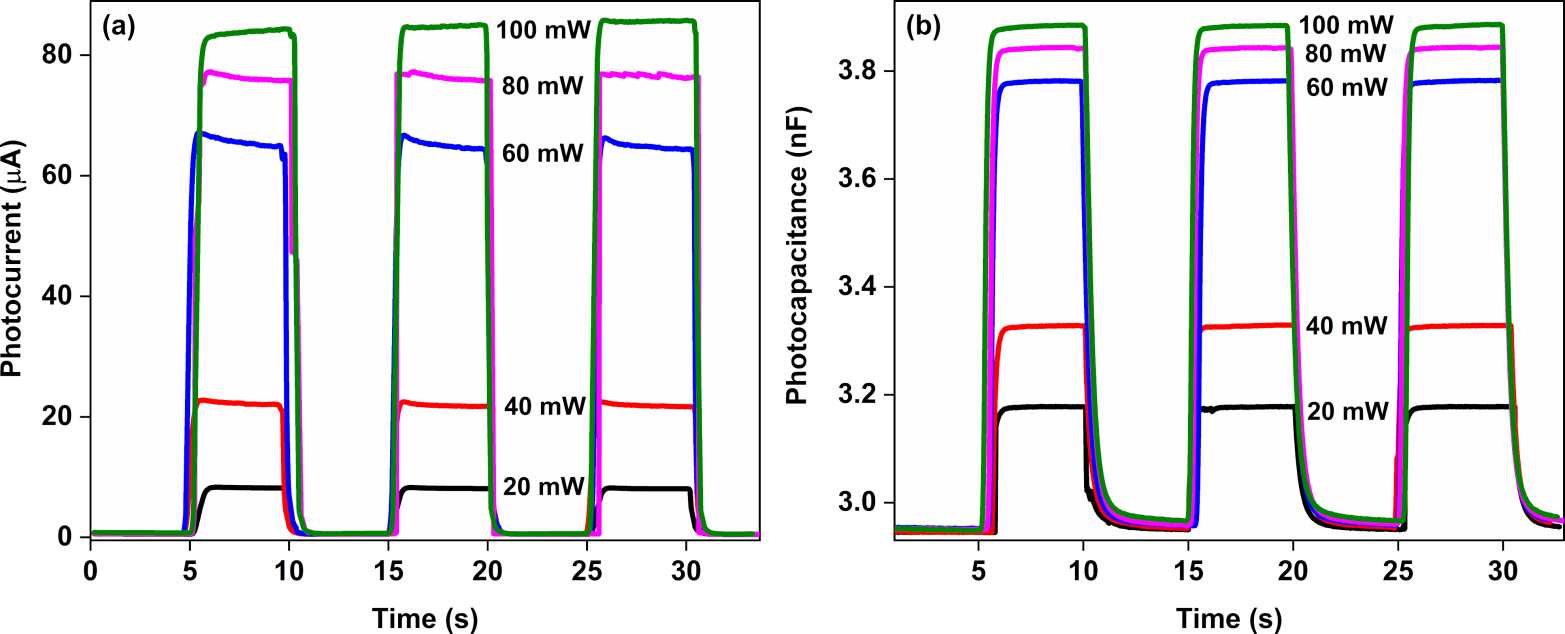
a) Photocurrent and b) photocapacitance changes of the Au/CuNiCoS_4_/p-Si photodiode under different power illumination densities.

Photocurrent and photocapacitance behavior of the Au/CuNiCoS_4_/p-Si photodiode have been tested by transient measurements while switching the light on and off for different illumination power densities. The photocurrent transient results are given in Figure 13a, the photocapacitance transient graphs are indicated in Figure 13b. The photodiode shows good response regarding both photocurrent and photocapacitance values. The photocurrent and photocapacitance values increased non-linearly with increasing illumination power density.

## Conclusion

CuNiCoS_4_ nanocrystals were synthesized successfully by hot injection and tested as interfacial layer in a metal semiconductor heterostructure. The XRD pattern of the CuNiCoS_4_ nanocrystals confirmed the crystal structure of the CuNiCoS_4_ nanocrystals with the cubic *Fd*−3*m* (227) space group. The HR-TEM and FE-SEM images revealed that CuNiCoS_4_ nanocrystals are spherical with interplanar distances of 2.87 Å. The EDX analysis confirmed the composition of the CuNiCoS_4_ nanocrystals. The CuNiCoS_4_ nanocrystals were inserted between Au and p-Si to fabricate a Au/CuNiCoS_4_/p-Si photodiode and characterized by *I*–*V* and *C*–*V* measurements for different illumination power densities and frequencies, respectively. Ideality factor, barrier height, series and shunt resistance values, as well as responsivity and specific detectivity were calculated and discussed in detail in terms of increasing light power. The Au/CuNiCoS_4_/p-Si photodiode has an ideality factor of 1.06 and a barrier height of 0.81 eV, and the ideality factor values increase and barrier height values decrease with increasing light power. The device exhibited good rectifying behavior as well as good photodiode properties at reverse biases. The *C*–*V* characteristics of the Au/CuNiCoS_4_/p-Si photodiode revealed that the capacitance values as well as other electrical parameters extracted from *C^−^*^2^–*V* plots strongly changed depending on frequency and voltage. The results show that the fabricated Au/CuNiCoS_4_/p-Si photodiode can be used in optoelectronic applications and has the potential to open new opportunities for improving the device performance.
